# SARS-CoV-2 Omicron variant shows less efficient replication and fusion activity when compared with Delta variant in TMPRSS2-expressed cells

**DOI:** 10.1080/22221751.2021.2023329

**Published:** 2022-01-19

**Authors:** Hanjun Zhao, Lu Lu, Zheng Peng, Lin-Lei Chen, Xinjin Meng, Chuyuan Zhang, Jonathan Daniel Ip, Wan-Mui Chan, Allen Wing-Ho Chu, Kwok-Hung Chan, Dong-Yan Jin, Honglin Chen, Kwok-Yung Yuen, Kelvin Kai-Wang To

**Affiliations:** a State Key Laboratory for Emerging Infectious Diseases, Carol Yu Centre for Infection, Department of Microbiology, Li Ka Shing Faculty of Medicine, The University of Hong Kong, Pokfulam, Hong Kong Special Administrative Region, People’s Republic of China; b School of Biomedical Sciences, Li Ka Shing Faculty of Medicine, The University of Hong Kong, Pokfulam, Hong Kong Special Administrative Region, People’s Republic of China; c Department of Microbiology, Queen Mary Hospital, Hong Kong Special Administrative Region, People’s Republic of China

**Keywords:** Delta variant, viral replication, Omicron variant, TMPRSS2, SARS-CoV-2

## Abstract

The novel SARS-CoV-2 Omicron variant (B.1.1.529), first found in early November 2021, has sparked considerable global concern and it has >50 mutations, many of which are known to affect transmissibility or cause immune escape. In this study, we sought to investigate the virological characteristics of the Omicron variant and compared it with the Delta variant which has dominated the world since mid-2021. Omicron variant replicated more slowly than the Delta variant in transmembrane serine protease 2 (TMPRSS2)-overexpressing VeroE6 (VeroE6/TMPRSS2) cells. Notably, the Delta variant replicated well in Calu3 cell line which has robust TMPRSS2 expression, while the Omicron variant replicated poorly in this cell line. Competition assay showed that Delta variant outcompeted Omicron variant in VeroE6/TMPRSS2 and Calu3 cells. To confirm the difference in entry pathway between the Omicron and Delta variants, we assessed the antiviral effect of bafilomycin A1, chloroquine (inhibiting endocytic pathway), and camostat (inhibiting TMPRSS2 pathway). Camostat potently inhibited the Delta variant but not the Omicron variant, while bafilomycin A1 and chloroquine could inhibit both Omicron and Delta variants. Moreover, the Omicron variant also showed weaker cell–cell fusion activity when compared with Delta variant in VeroE6/TMPRSS2 cells. Collectively, our results suggest that Omicron variant infection is not enhanced by TMPRSS2 but is largely mediated via the endocytic pathway. The difference in entry pathway between Omicron and Delta variants may have an implication on the clinical manifestations or disease severity.

## Introduction

Severe acute respiratory syndrome coronavirus 2 (SARS-CoV-2) has been associated with more than 5 million deaths worldwide. Efficient person-to-person transmission, contributed by a high viral load in upper respiratory tract secretion at the early phase of infection [1], allowed SARS-CoV-2 to emerge and spread among humans [2]. SARS-CoV-2 efficiently uses several human host factors for viral attachment and entry. The spike protein of SARS-CoV-2 can attach to the host cell receptor human angiotensin-converting enzyme 2 (ACE2) more efficiently than that from closely related coronaviruses [3]. Soluble ACE2 is important for entry in a human kidney cell line [4]. Furthermore, SARS-CoV-2 uses human heparan sulphate, C-type lectin receptors, DC-SIGN, L-SIGN, and sialic acid-binding Ig-like lectin 1 (SIGLEC1) as attachment factors [5,6]. Human transmembrane serine protease 2 (TMPRSS2) and endosomal proteases cathepsins are required for the activation of the spike protein to facilitate membrane fusion [7]. Our previous study using antivirals to target both TMPRSS2- and endocytic-pathways resulted in a potent antiviral effect [8].

The continual global circulation of SARS-CoV-2 is fuelled by the appearance of variants, especially those with key mutations in the spike protein receptor-binding domain (RBD). Several of these new variants, especially those classified as Variants of Concern (VOCs) by the World Health Organization, can transmit more easily [9]. Our previous study showed that the higher transmissibility of the Alpha variant correlated with a lower infectious dose in hamsters [10]. Furthermore, these VOCs have reduced susceptibility to neutralizing antibodies induced by natural COVID-19 infection or first-generation COVID-19 vaccines and have led to reinfections or vaccine breakthrough infections [11–13].

A novel SARS-CoV-2 lineage B.1.1.529, first reported by South Africa and Botswana, has been classified by the World Health Organization as a VOC on 26th November 2021 [14]. This novel variant, now known as the Omicron variant, is particularly worrisome because of the unusually large number of mutations, including those that are known to cause escape from neutralizing antibodies and increased binding to the host cell receptor angiotensin-converting enzyme-2 (ACE2). Furthermore, epidemiological investigation in South Africa showed that the Omicron variant is associated with a higher risk of reinfection [15] and a faster doubling time [16]. In Hong Kong, a patient with Omicron variant infection was believed to have been transmitted via the airborne route within a designated quarantine hotel [17]. In this study, we assessed the risks of the Omicron variant by comparing the viral replication and fusion activity with the Delta variant, which has dominated the world since May 2021.

## Methods

### Virus

The SARS-CoV-2 Omicron variant strain was isolated from the combined nasopharyngeal throat swab of a COVID-19 patient in Hong Kong (hCoV-19/Hong Kong/HKU-344/2021; GISAID accession number EPI_ISL_7357684). The Delta variant isolate (B.1.617.2) (hCoV-19/Hong Kong/HKU-210804-001/2021; GISAID accession number EPI_ISL_3221329) has been described previously [18].

### Viral culture

The viral culture was performed in a biosafety level 3 facility as we described previously [12]. Briefly, TMPRSS2-expressing VeroE6 (VeroE6/TMPRSS2) cells (JCRB Cat^#^JCRB1819) were seeded with 100 μL of minimum essential medium (MEM) (Thermo Fisher Scientific) at 4 × 10^4^ cells in a 96-well plate and incubated at 37°C in a carbon dioxide incubator until confluence for inoculation. Each well was inoculated with 30 μL of clinical specimen. The virus-induced cytopathic effect was examined. Cultures with more than 50% virus-induced cytopathic effect were expanded to large volume, and 50% tissue culture infective dose (TCID_50_) was determined. The whole-genome sequence of the culture isolates was confirmed using nanopore sequencing as we described previously [13].

### Viral replication kinetics

VeroE6/TMPRSS2, parental VeroE6, and Calu3 cells were seeded on a 96-well plate for overnight culture. After removing culture media and washing cells by phosphate-buffered saline, SARS-CoV-2 (0.1 TCID_50_ of Omicron or Delta variant) was inoculated to cells for infection at 37°C for 1 h. After removing the non-infectious virus, fresh media were added to cells for viral culture. Cell culture supernatants were collected at 1, 12, 24, 48, and 72 hpi, and viral RNA was extracted for RT-qPCR assay to determine the viral titres.

### RT-qPCR assay

Viral RNA was extracted by Viral RNA Mini Kit (QIAGEN, Cat^#^ 52906, USA) according to the manufacturer’s instructions. Extracted RNA was reverse transcribed to cDNA using PrimeScript II 1st Strand cDNA synthesis Kit (Takara, Cat^#^ RR036A) using GeneAmp® PCR system 9700 (Applied Biosystems, USA). The cDNA was then amplified using specific spike primers [8] for detecting SARS-CoV-2 using LightCycler® 480 SYBR Green I Master (Roach, USA). For quantitation, 10-fold serial dilutions of standard plasmid equivalent to 10^1^–10^6^ copies per reaction were prepared to generate the calibration curve. Real-time qPCR experiments were performed using LightCycler® 96 system (Roche, USA).

### Fusion assay

VeroE6/TMPRSS2 cells were seeded on 96-well plates for overnight culture. The GFP plasmid was packaged by Lipofectamine 3000 (Invitrogen, Cat^#^ L3000008) and transfected to cells in each well. At 8 h post-transfection, 0.1 TCID_50_ virus was added to GFP-transfected cells. At 24, 48, and 72 hpi, cell images were taken by fluorescence microscope. GFP-transfected cells without viral infection were the negative control of virus-induced cell–cell fusion. Images were taken from two independent biological samples.

### Competition assay

Whole-genome sequencing with tiling PCR was performed using nanopore sequencing. The percentage of reads corresponding to the Delta and Omicron variant at the spike amino acid residues 222 (Omicron: A; Delta: V), 655 (Omicron: Y; Delta H), and 950 (Omicron: D, Delta: N) was used for the determination of competition results.

### Statistical analysis

The statistical significances of viral replication in cells were calculated by the two-tailed Student’s *t*-test. A *P* value of <0.05 was considered to be statistical.

## Results

### Omicron variant replication is less efficient than Delta variant in VeroE6/TMPRSS2 or Calu3 cells

To compare the viral replication kinetics of the Omicron and Delta variants, we infected VeroE6/TMPRSS2 cells, non-TMPRSS2-expressing parental VeroE6 cells or Calu3 cells with 0.1 TCID_50_ of Omicron or Delta variants, and viral RNA copies were measured up to 72 hpi.

**Figure 1.**
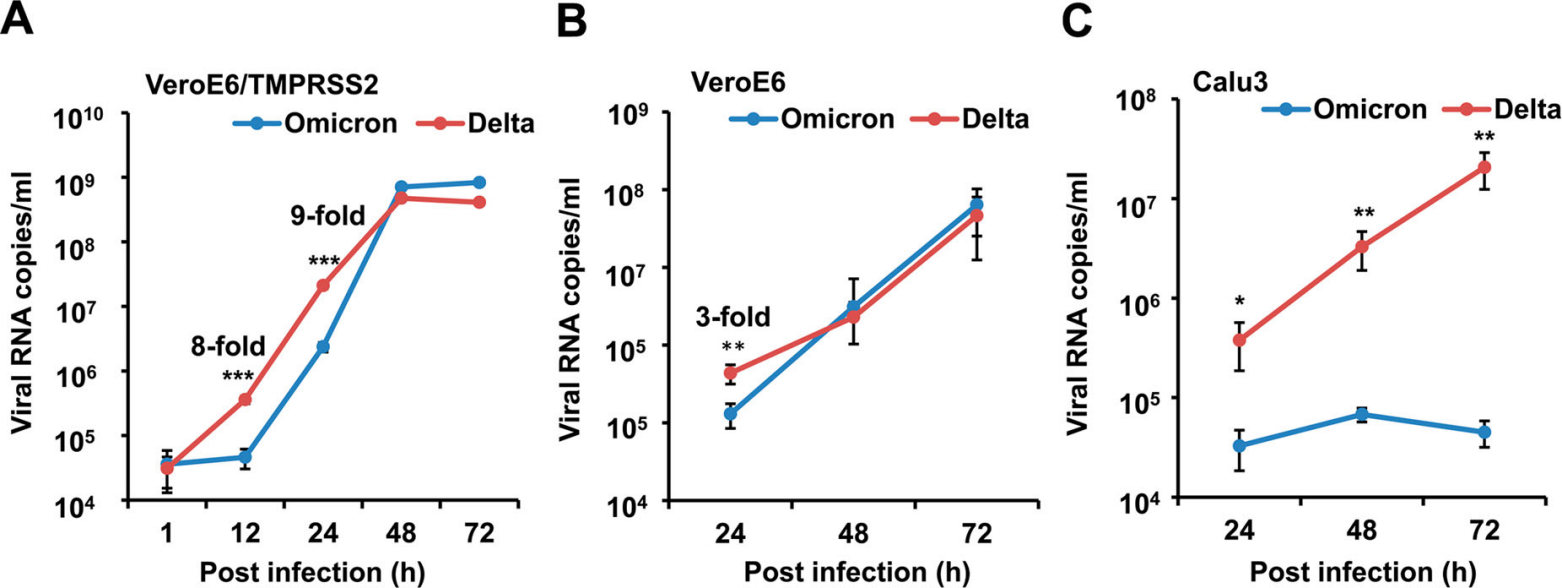
Viral replication in VeroE6/TMPRSS2, VeroE6 and Calu3 cells. (A) Viral replication kinetics in VeroE6/TMPRSS2 cells. (B) Viral replication kinetics in VeroE6 cells. (C) Viral replication kinetics in Calu3 cells. SARS-CoV-2 (0.1 TCID_50_ of Omicron or Delta variant) was inoculated to cells for viral infection. Cell culture supernatants were collected at indicated time points for RT-qPCR assay to determine viral RNA titres. Data are presented as mean ± SD from two independent experiments with four biological samples. * indicates *P *< 0.05, ** indicates *P *< 0.01 and *** indicates *P *< 0.001 when compared with Omicron variant.

In VeroE6/TMPRSS2 cells, the Omicron variant had significantly lower viral loads at 12 hpi (4.6 × 10^4^ copies/ml vs 3.6 × 10^5^ copies/ml, *P *< 0.001) and 24 hpi (2.4 × 10^6^ copies/ml vs 2.2 × 10^7^ copies/ml, *P *< 0.001) than that of the Delta variant (Figure 1(A)). In VeroE6 cells, the difference in viral titres between Omicron and Delta variant at 24, 48, and 72 hpi (Figure 1(B)) were less than three folds. In Calu3 cells with robust TMPRSS2 expression, the Omicron variant had a significantly lower viral load than that of the Delta variant from 24 hpi to 72 hpi (Figure 1(C)). Taken together, these results suggested that the replication of the Omicron variant is less efficient than Delta variant in VeroE6/TMPRSS2 or Calu3 cells.

### The Delta variant outcompetes Omicron variant in VeroE6/TMPRSS2 cells but not parental VeroE6 cells

**Figure 2.**
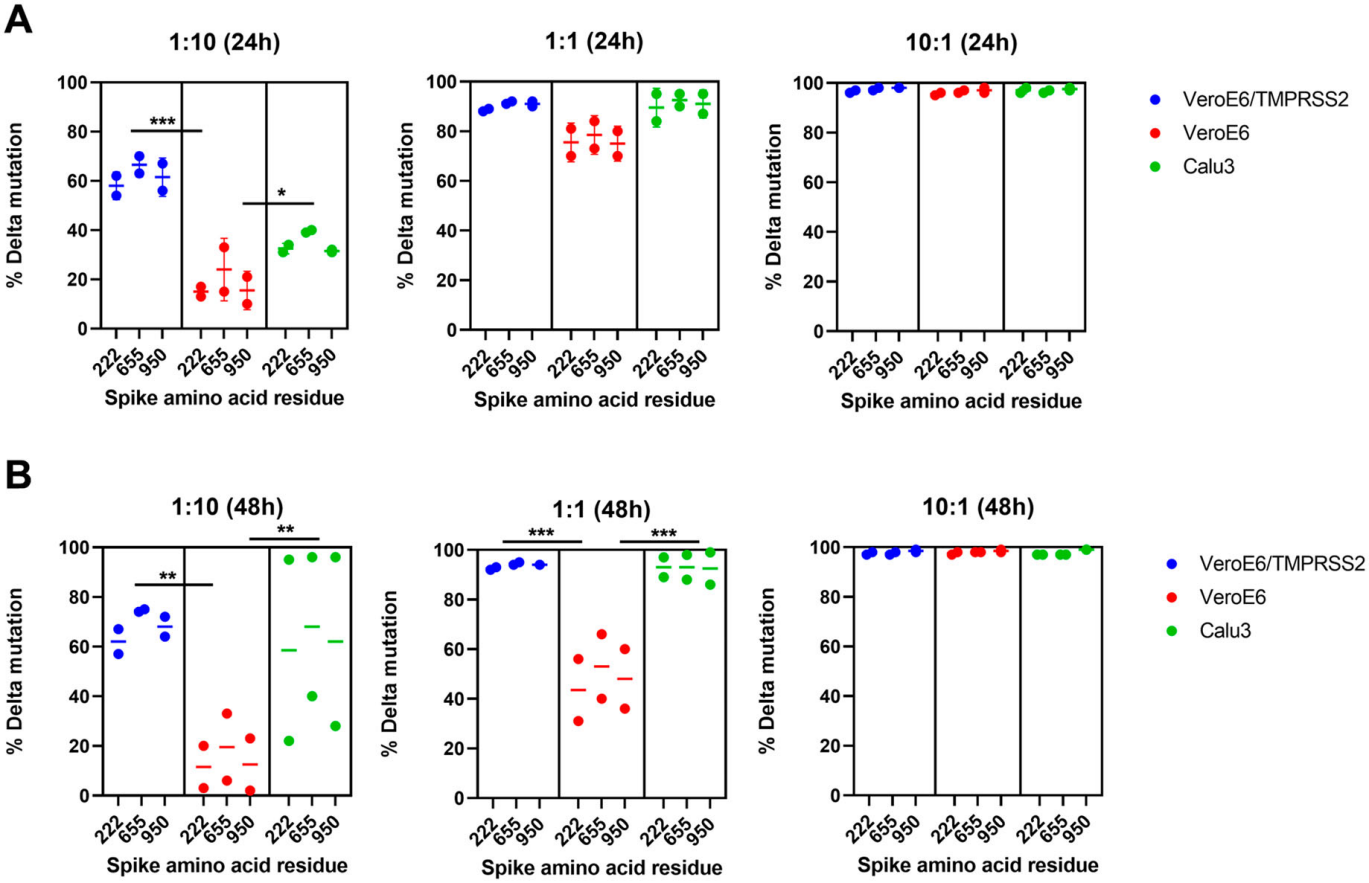
Delta variant outcompeted the replication of Omicron variant in VeroE6/TMPRSS2 and Calu3 cells. VeroE6/TMPRSS2, VeroE6, and Calu3 cells were co-infected by the Delta and Omicron variants with the Delta:Omicron ratio of 1:10, 1:1 and 10:1. Cell culture supernatants were collected at 24 hpi (A) and 48 hpi (B) for next-generation sequencing. The percentage of reads corresponding to the Delta and Omicron variant is determined for spike amino acid residues 222 (Omicron: A; Delta: V), 655 (Omicron: Y; Delta H), and 950 (Omicron: D, Delta: N). * indicates *P *< 0.05, ** indicates *P *< 0.01 and *** indicates *P *< 0.001.

To better assess the difference in fitness between Omicron and Delta variants with or without over-expression of TMPRSS2, we performed a competition assay for Omicron and Delta variants in parental VeroE6, VeroE6/TMPRSS2 and Calu3 cells. We used nanopore sequencing to quantitate the percentage of each variant based on the percentage of reads at three different positions that differ between Delta and Omicron variants (spike protein amino acid residue 222, 655, and 950). At a Delta:Omicron ratio of 1:10, Delta variant outcompeted Omicron variant in VeroE6/TMPRSS2 and Calu3 cells but not VeroE6 cells (Figure 2). Our results confirmed that the Delta variant has a much better fitness than Omicron variant in the presence of TMPRSS2.

### Omicron variant replication is dependent on endocytosis but not TMPRSS2

**Figure 3.**
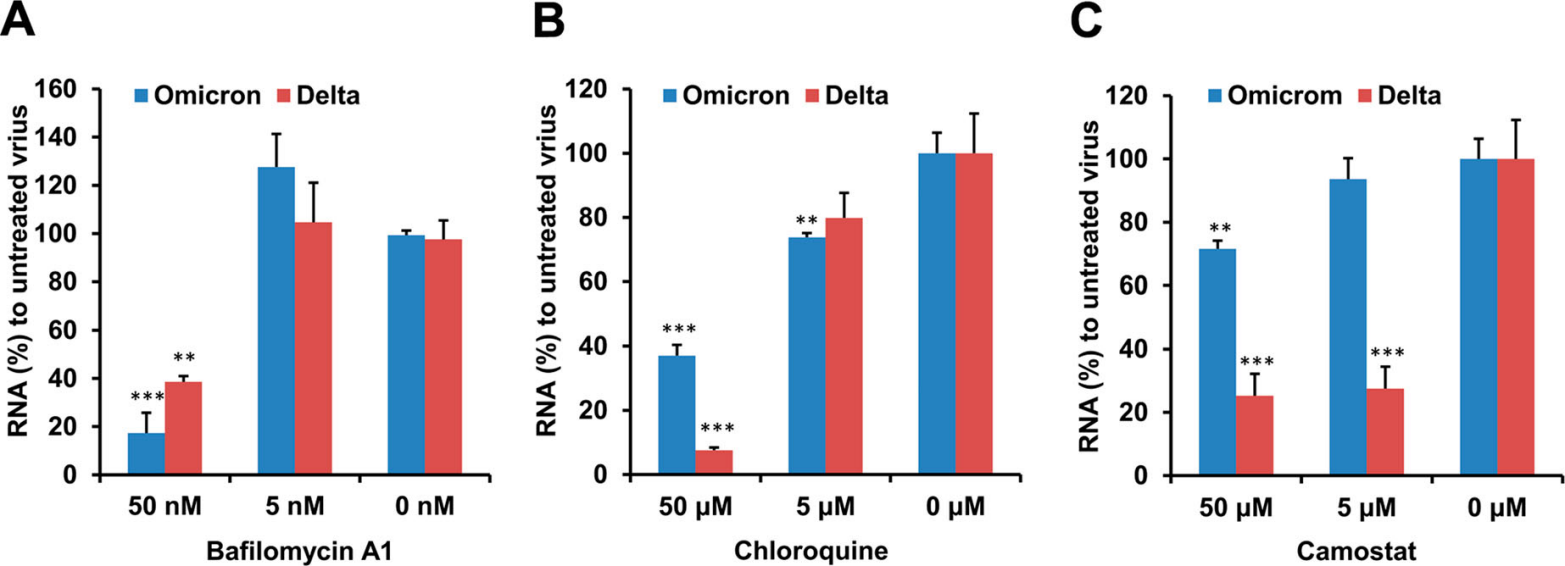
Camostat could not potently inhibit the replication of Omicron variant in VeroE6/TMPRSS2 cells. (A) Bafilomycin A1 inhibited the Omicron and Delta replication in VeroE6/TMPRSS2 cells. (B) Chloroquine inhibited the Omicron and Delta replication in VeroE6/TMPRSS2 cells. (C) Camostat could potently inhibit Delta variant but not Omicron variant replication in VeroE6/TMPRSS2 cells. Cells were pretreated by the indicated bafilomycin A1, chloroquine or camostat before viral infection, 1000 TCID_50_ of virus was added to cells for infection and then viral RNA copies in cell lysate were measured at 8 hpi by RT-qPCR. Viral RNA (%) was normalized to the viral RNA copy of untreated virus. ** indicates *P *< 0.01 and *** indicates *P *< 0.001 when compared with untreated virus. Data are presented as mean ± SD from three independent biological samples.

To confirm whether Omicron variant infection was mainly dependent on endocytosis but not TMPRSS2 pathway, we compared the effect of bafilomycin A1, chloroquine (an endocytosis inhibitor) and camostat (a TMPRSS2 inhibitor) on the replication of Omicron and Delta variants. Bafilomycin A1 and chloroquine could significantly inhibit both Omicron and Delta variant viral replication in VeroE6/TMPRSS2 cells (Figure 3(A,B)). In contrast, the inhibition by camostat was much less potent for the Omicron variant than that of the Delta variant (Figure 3(C)). These results demonstrated that the Omicron variant mainly depended on the endocytic pathway but not the TMPRSS2 pathway for viral replication.

### Omicron variant shows weak fusion activity in VeroE6/TMPRSS2 cells

**Figure 4.**
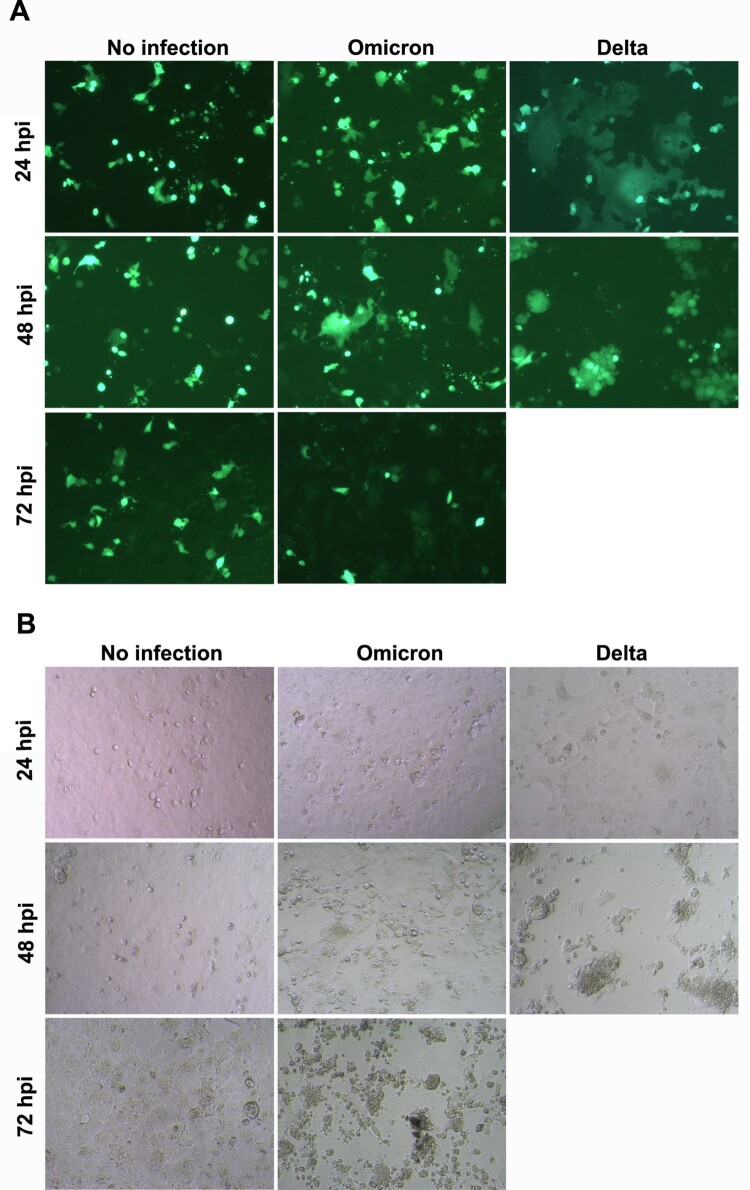
SARS-CoV-2 mediated cell-cell fusion. (A) Cell GFP images of infected cells at indicated post infection time. (B) Bright field images of infected cells at indicated post infection time. VeroE6/TMPRSS2 cells were transfected with GFP plasmid and infected with 0.1 TCID_50_ virus (Omicron and Delta variant). Images were taken at 24, 48, and 72 hpi. GFP-transfected cells without viral infection were the negative control of cell fusion (no infection). Scale bar = 50 μm. Experiment images were taken from two independent biological samples.

Next, we compared the fusion activity between Omicron and Delta variants in VeroE6/TMPRSS2 cells. At 24 hpi, the Delta variant-infected cells showed large cell–cell fusion (fusion cell sizes >200 μm), while the Omicron variant-infected cells did not show cell–cell fusion (Figure 4(A)), in which cell sizes were similar as the non-fusion cell control (cell sizes: ∼20 μm). At 24 hpi, there was no obvious CPE being observed in both Delta and Omicron variant infected cells (Figure 4(B)). At 48 hpi, the Delta variant infected cells were almost detached with significant CPE (>90%) when compared with non-infected cells. However, the Omicron variant only showed very few cell–cell fusion, in which cell sizes were less than 100 μm, and no significant CPE (<10%) was observed. At 72 hpi, Delta variant-infected cells showed 100% CPE, while Omicron variant-infected cells showed ∼80% CPE. However, there was no large cell–cell fusion for Omicron variant (Figure 4(A)). Hence, the Omicron variant had a much weaker fusion activity than the Delta variant in VeroE6/TMPRSS2 cells.

## Discussions

The World Health Organization classified the Omicron variant as a VOC only 2 days after being notified by the South African scientists, mainly based on the unusually large number of amino acid mutations in the spike protein and the rapid increase in South Africa. It is worrisome that the Omicron variant may transmit more easily and may replace the Delta variant which has dominated the World up to November 2021. Here, we found that the replication and fusion activity of the Omicron variant is much less dependent on TMPRSS2, while the replication and fusion of the Delta variant is greatly enhanced in VeroE6/TMPRSS2 cells.

Conformational change of the spike protein is essential in mediating the viral and cellular membrane fusion [19], which in turn allows the viral genome to reach the cytoplasm via a fusion pore. After cleavage of the S1/S2 junction by furin, the S2’ site can be cleaved by either the cell surface TMPRSS2 or the endosomal cathepsin B/L. A single-cell sequencing study showed that TMPRSS2 is highly expressed in alveolar type I and type II cells of the lung [20]. The enhancement of viral replication of the Delta variant by TMPRSS2 corroborates with previous animal studies showing more extensive infection of alveolar pneumocytes for the Delta variant [21]. In the current study, we showed that in contrast to the Delta variant, Omicron variant was not effective in using TMPRSS2 for viral replication. Our results suggest that the Omicron variant may have poorer replication in the lungs when compared with the Delta variant. Indeed, there are preliminary epidemiological studies showing that the Omicron variant may have the milder disease [22].

Although there is a marked difference in the dependence of TMPRSS2 on viral replication, there is no difference in the S2’ cleavage site between the Omicron and Delta variants. The difference in TMPRSS2 dependence may be related to the furin cleavage site, in which the Omicron variant is P681H and the Delta variant is P681R. Using a pseudovirus system, Peacock et al. have demonstrated that the TMPRSS2-mediated entry is much greater in pseudovirus carrying the polybasic furin cleavage site at the S1/S2 junction than those with the polybasic cleavage site deletion [23]. We showed that the Omicron variant is much less fusogenic when compared with the Delta variant. In addition to the replication difference in TMPRSS2 dependence, the poorer fusion activity of the Omicron variant can be attributed to the difference in the S1/S2 junction furin cleavage site. Previous studies have demonstrated that the Delta variant exhibited higher fusion activity than the wild type virus or the Alpha variant that contains P681H mutation [21,24,25]. Since syncytia formation is found in postmortem lung specimens of deceased COVID-19 patients, the fusion activity may be associated with disease severity [26].

In addition to being a protease that cleaves the spike protein for activation, TMPRSS2 also plays a role as an interferon antagonist. Overexpression of TMPRSS2 can reverse the restriction of SARS-CoV-2 replication by NCOA7, an interferon-stimulated gene [27]. It would be important to further assess how this innate immune role of TMPRSS2 will affect viral replication.

Although both VeroE6/TMPRSS2 and Calu3 exhibit high levels of TMRPSS2 expression, there was a difference in the replication of the Omicron variant between these two cell lines. While the Omicron variant could replicate to a level similar to Delta variant at 48 and 72 hpi in VeroE6/TMPRSS2 cells, the viral load of the Omicron variant remained significantly lower than the Delta variant at 48 and 72 hpi in Calu3 cells. This may be because the endocytosis is not an entry pathway for SARS-CoV-2 in Calu3 cells. Hoffmann et al. have shown that chloroquine, an endosomal acidification inhibitor, did not inhibit SARS-CoV-2 replication in Calu3 cells [28]. In Figure 3, we demonstrated that chloroquine could inhibit the replication of SARS-CoV-2, suggesting that the virus can enter VeroE6/TMPRSS2 cells by endosomal pathway.

At the time of writing, the Omicron variant has already affected over 90 countries worldwide. Our *in vitro* study provides a rapid phenotypic characterization of the Omicron variant which is important for the urgent public health risk assessment. It remains to be confirmed in animal models whether the difference in TMPRSS2 dependence will lead to the difference in disease severity or tissue tropism.
